# Thermoelastic State of a Magnetocaloric Ferromagnetic Plate Under Constant-Rate Ambient Temperature Rise

**DOI:** 10.3390/ma19163544

**Published:** 2026-08-21

**Authors:** Roman Musii, Myroslava Klapchuk, Uliana Zhydyk, Nelya Pabyrivska, Zenoviy Kohut, Dariusz Całus, Piotr Gębara, Karolina Kutynia

**Affiliations:** 1 Institute of Applied Mathematics and Fundamental Sciences, Lviv Polytechnic National University, 79013 Lviv, Ukraine; roman.s.musii@lpnu.ua (R.M.); myroslava.i.klapchuk@lpnu.ua (M.K.); uliana.v.zhydyk@lpnu.ua (U.Z.); nelia.v.pabyrivska@lpnu.ua (N.P.); zinoviy.o.kohut@lpnu.ua (Z.K.); 2 Faculty of Electrical Engineering, Czestochowa University of Technology, 42-200 Czestochowa, Poland; dariusz.calus@pcz.pl; 3 Faculty of Production Engineering and Materials Technology, Czestochowa University of Technology, 42-201 Czestochowa, Poland; karolina.kutynia@pcz.pl

**Keywords:** ferromagnetic plate, magnetocaloric material, carbon steel, surface heating, temperature, stress

## Abstract

A simply supported rectangular isotropic ferromagnetic plate with magnetocaloric properties subjected to convective heating on its upper surface is considered. The governing equations comprise a system of two-dimensional transient equations for the thickness-averaged temperature characteristics of the plate and the two-dimensional bending equations for isotropic plates expressed in terms of generalized displacements within the framework of first-order shear deformation theory. Closed-form solutions to the governing equations are obtained by expanding all thermal and mechanical field quantities in double Fourier sine series satisfying the prescribed boundary conditions, combined with the Laplace transform in time applied to the thermal quantities. A comparative numerical analysis is carried out for the magnetocaloric ferromagnetic plate and a carbon steel plate under ambient temperature rising at a finite rate to a prescribed value. The dependences of all quantities under investigation on the ambient temperature rise rate, time, convective heat transfer coefficient, thermal conductivity of the ferromagnetic material, and geometric parameters of the plate are analyzed and presented graphically. The results obtained provide a quantitative basis for assessing the thermoelastic state and for optimizing the geometry and operating conditions of active magnetic regenerator plate stacks with a view to enhancing their structural reliability.

## 1. Introduction

In recent years, the growth in global energy consumption and environmental safety requirements have led to an active search for alternative cooling technologies. One of the most promising areas is magnetic cooling based on the magnetocaloric effect (MCE)—a fundamental thermodynamic phenomenon in which a ferromagnetic material adiabatically changes its temperature under the influence of an alternating magnetic field. Unlike traditional vapor-compression systems, magnetic cooling does not require gaseous refrigerants, making it an environmentally friendly alternative for household refrigerators and heat pumps.

A wide range of magnetocaloric materials (MCMs) has been considered for use in cooling devices [1]. Materials with a first-order phase transition attract particular attention, as they are characterized by an extremely high change in magnetic entropy $\Delta S_{M}$ within a narrow temperature range near the transition. The most important representative of this class is the intermetallic compound La(Fe,Si)_13_H_*y*_, doped with various elements in the Fe and Si sites [2]. This series of alloys is characterized by high MCE, a tunable Curie temperature, and minimal thermal hysteresis. The paper [3] provides a systematic review of the MCE and thermomagnetic properties of selected MCMs—in particular, Gd-based, MnCoGe-based, and La(Fe,Si)_13_—with prospects for synthesizing new materials with modified chemical compositions to achieve optimized properties for active magnetic regenerators (AMRs) operating near room temperature.

A series of experimental studies [4,5] is devoted to the detailed characterization of the thermophysical and magnetic properties of LaFeCoSi-type MCMs. In particular, the work [4] established a negative lattice expansion of the La(Fe,Si)_13_ phase in the LaFe_11.0_Co_0.7_Si_1.3_ alloy modified with Ce, Ho, Pr, or Mn: a gradual decrease in the relative change in the lattice parameter with increasing Mn content was observed, as well as an anomalous temperature dependence of the lattice constant for the α-Fe phase, caused by the negative expansion of the La(Fe,Si)_13_ phase. Changes in entropy and the regulation of the Curie temperature for different chemical compositions have been analyzed in detail in the work [5]. In particular, the critical behavior of the LaFe_11.0_Co_0.7_Si_1.3_ alloy near the Curie point was investigated, the calculated critical parameters indicate long-range ferromagnetic interactions described by mean-field theory.

Despite significant progress in the study of the magnetic and thermal properties of MCM, a key obstacle to their commercialization remains the low mechanical stability of La(Fe,Mn,Si)_13_ alloys under the combined action of thermal, magnetic, and mechanical loads during manufacturing and cyclic operation of devices [6]. Fabricating an efficient heat exchanger with a specified geometry from such materials and ensuring the necessary cyclic stability is a complex engineering challenge. In particular, the fracture toughness—the key parameter characterizing a material’s resistance to crack propagation—has not yet been determined for the matrix phase of these alloys. The practical significance of this problem is confirmed by studies of crack initiation in La(Fe,Mn,Si)_13_ plates [7], where the four-point bending method was used on specimens with a fixed defect size to obtain a residual strength curve that quantitatively links the observed defects to the mechanical strength of the material. The work [8] shows that the chemical interaction of La_2_O_3_ particles with moisture causes volumetric expansion and internal degradation, which initiates crack initiation in the 1:13 phase near La-enriched inclusions. The work [9], in turn, demonstrates the practical application of plates made of bonded La(Fe,Si,Mn)_13_H_*y*_ as an AMR in a small-scale magnetocaloric device: under optimal operating conditions, a temperature difference of 6.4 K was achieved across the plates; however, the long-term mechanical stability of the plates remains an open question.

Thus, the available experimental data clearly indicate the need to develop analytical tools for predicting the thermoelastic behavior of laminated MCM elements at the design stage. A key feature of this problem is that ferromagnetic alloys of the type La(Fe,Si)_13_ exhibit an anomalous—negative and significantly nonlinear—temperature dependence of the coefficient of thermal expansion α near the Curie point, which qualitatively distinguishes them from structural materials, in particular, carbon steel, for which α is practically constant within the operating temperature range.

The papers [10,11] lay the groundwork for studying the stress–strain state of thin-walled structures under thermal and electromagnetic loads. Analytical solutions based on two-dimensional equations of classical and various refined theories have been obtained, particularly in the works [12,13,14]. The behavior of composite plates under conditions of loss of thermal stability was studied in the work [15], as well as in more recent works on three-layer/functionally graded plates under thermomechanical loading, where analytical solutions for displacements and stresses in simply supported rectangular functionally graded plates were obtained using higher-order theory [16,17].

It follows from the above review that the issue of the thermoelastic behavior of plates made of ferromagnetic materials with MCE, under the influence of external temperature factors, has not been sufficiently addressed in the literature. It is known that in a real active magnetic regenerator (AMR), the operating cycle consists of alternating magnetization and demagnetization phases, separated by heat exchange phases, during which the magnetocaloric plates transfer thermal energy to and from the coolant flowing through the regenerator channels. It is this heat exchange phase—convective heat exchange between the plate surface and the working fluid—that is modeled and investigated in this work. The temperature of the external environment, which increases with a finite rate to a given value $t^{*}$, physically represents the temperature of the coolant fluid during the heat exchange phase between the plate surface and the working fluid. Accordingly, the parameter $\beta^{*}$ characterizes the rate at which the plate surface approaches the temperature of the fluid. The value of $t^{*}$ is naturally related to the adiabatic temperature change $\Delta T_{ad}$ of the magnetocaloric material, which is set by the previous magnetization stage.

This work does not consider the full magneto-thermo-mechanical coupling, but only investigates one separate stage of the AMR cycle—the thermal stress state that develops in the plate during convective heat transfer—using the thermophysical properties of the magnetocaloric alloy (in particular, its anomalous coefficient of thermal expansion near the Curie point) as input parameters [3,4]. The purpose of this work is to conduct an analytical study of the distributions of the temperature field, normal displacements, and stresses in a rectangular isotropic ferromagnetic plate with MCE and freely supported edges, heated by the surrounding environment through its front surface, and to compare the obtained results with similar characteristics of a carbon steel plate. Graphical dependencies were obtained for various values of the surface temperature rise rate β∗, the heat transfer coefficient Bi, the thermal conductivity coefficient of the ferromagnetic material λ, and the geometric parameters of the plate. A comparative analysis aims to quantitatively reveal the contrast between the anomalous behavior of the MCM and the monotonic response of structural steel, which is a necessary basis for assessing the risk of failure and optimizing the geometry of AMR device plates.

## 2. Problem Formulation

Consider a rectangular plate of dimensions a×b and uniform thickness 2h, made of a homogeneous isotropic material. In the Cartesian coordinate system Oxyz, the plate occupies the domain [0,a]×[0,b]×[−h,h].

The plate surfaces z=±h are exposed to a surrounding medium whose temperatures are prescribed as

$$t_{z}^{\pm }\left(x,y,\tau \right)=t_{c}^{\pm }\left(x,y\right)\;t^{\pm }\left(\tau \right).$$

Convective heat exchange between each surface and the medium is governed by the heat transfer coefficients $\alpha^{+}$ and $\alpha^{-}$, respectively. The transient temperature field t(x,y,z,τ) satisfies the three-dimensional heat conduction equation [11].

To reduce the problem to two spatial dimensions, the temperature distribution across the thickness is approximated as linear in *z*-direction [18,19]:

(1) $$t\left(x,y,z,\tau \right)=T_{1}\left(x,y,\tau \right)+\frac{z}{h}\;T_{2}\left(x,y,\tau \right),$$

where the thickness-averaged characteristics $T_{1}$ and $T_{2}$ are defined by

$$T_{1}=\frac{1}{2h}\int_{-h}^{h}t\;dz,\;T_{2}=\frac{3}{2h^{2}}\int_{-h}^{h}t\;z\;dz.$$

Integrating the heat conduction equation over the thickness coordinate *z* and applying these definitions yields the following system of coupled two-dimensional equations for $T_{1}$ and $T_{2}$:

(2) $$\begin{array}{cc} \Delta T_{1}-\varepsilon_{1}^{t}\;T_{1}-\varepsilon_{2}^{t}\;T_{2}-\frac{1}{\kappa }\frac{\partial T_{1}}{\partial \tau }\; & =-f_{1},\\ \Delta T_{2}-3\varepsilon_{2}^{t}\;T_{1}-3\;\left(\frac{1}{h^{2}}+\varepsilon_{1}^{t}\right)T_{2}-\frac{1}{\kappa }\frac{\partial T_{2}}{\partial \tau }\; & =-f_{2}. \end{array}$$

Here $\Delta =\partial^{2}/\partial x^{2}+\partial^{2}/\partial y^{2}$ is the two-dimensional Laplacian, $\kappa =\lambda /c_{v}$ is the temperature conductivity, λ is the thermal conductivity, $c_{v}$ is the volumetric heat capacity, and τ denotes time. The expressions are accordingly

$$\varepsilon_{1}^{t}=\frac{\alpha^{+}+\alpha^{-}}{2h\lambda },\;\varepsilon_{2}^{t}=\frac{\alpha^{+}-\alpha^{-}}{2h\lambda },$$

$$t_{1}^{z}=\frac{1}{2}\left(t_{z}^{+}+t_{z}^{-}\right),\;t_{2}^{z}=\frac{1}{2}\left(t_{z}^{+}-t_{z}^{-}\right),$$

$$f_{1}=\varepsilon_{1}^{t}\;t_{1}^{z}+\varepsilon_{2}^{t}\;t_{2}^{z}=f_{1}^{z}\left(x,y\right)\;f_{1}^{\tau }\left(\tau \right),\;f_{2}=3\left(\varepsilon_{2}^{t}\;t_{1}^{z}+\varepsilon_{1}^{t}\;t_{2}^{z}\right)=f_{2}^{z}\left(x,y\right)\;f_{2}^{\tau }\left(\tau \right).$$

The stress–strain state of the plate is governed by the first-order shear deformation theory (FSDT) [14,20] whose bending equations for a homogeneous isotropic plate are written in terms of three generalized kinematic unknowns—the transverse deflection *w* and the rotations of the transverse normal $\gamma_{1}$, $\gamma_{2}$ about the *y*- and *x*-axes, respectively:

(3) $$\begin{array}{cc} \; & \Delta w+\frac{\partial \gamma_{1}}{\partial x}+\frac{\partial \gamma_{2}}{\partial y}=0,\\ \; & \frac{h^{2}}{3}\;\left(\frac{2}{1-\nu }\frac{\partial^{2}\gamma_{1}}{\partial x^{2}}+\frac{\partial^{2}\gamma_{1}}{\partial y^{2}}\right)+\frac{h^{2}}{3}\;\frac{1+\nu }{1-\nu }\frac{\partial^{2}\gamma_{2}}{\partial x\;\partial y}-k^{\prime }\;\left(\gamma_{1}+\frac{\partial w}{\partial x}\right)=\frac{2h}{3}\;\frac{1+\nu }{1-\nu }\;\alpha_{t}\frac{\partial T_{2}}{\partial x},\\ \; & \frac{h^{2}}{3}\;\frac{1+\nu }{1-\nu }\frac{\partial^{2}\gamma_{1}}{\partial x\;\partial y}+\frac{h^{2}}{3}\;\left(\frac{\partial^{2}\gamma_{2}}{\partial x^{2}}+\frac{2}{1-\nu }\frac{\partial^{2}\gamma_{2}}{\partial y^{2}}\right)-k^{\prime }\;\left(\gamma_{2}+\frac{\partial w}{\partial y}\right)=\frac{2h}{3}\;\frac{1+\nu }{1-\nu }\;\alpha_{t}\frac{\partial T_{2}}{\partial y}, \end{array}$$

where ν is Poisson’s ratio; $\alpha_{t}$ is the coefficient of linear thermal expansion; $T_{2}$ is the antisymmetric thickness-averaged temperature characteristic defined in (1); and $k^{\prime }$ is the shear correction factor (here $k^{\prime }=5/6$), which accounts for the non-uniform distribution of transverse shear stresses across the thickness.

Normal stresses due to generalized displacements are determined using the following formulas:

$$\sigma_{x}=\frac{E}{1-\nu^{2}}\left[z\left(\frac{\partial \gamma_{1}}{\partial x}+\nu \frac{\partial \gamma_{2}}{\partial y}\right)-\left(1+\nu \right)\alpha_{t}t\right],$$

(4) $$\sigma_{y}=\frac{E}{1-\nu^{2}}\left[z\left(\frac{\partial \gamma_{2}}{\partial y}+\nu \frac{\partial \gamma_{1}}{\partial x}\right)-\left(1+\nu \right)\alpha_{t}t\right].$$

Systems (2) and (3) are closed by the simply supported boundary conditions on all four edges together with the initial conditions for the temperature field.

Edges x=0 and x=a  (y∈(0,b), τ>0):

(5) $$w=0,\;\gamma_{2}=0,\;M_{x}=D\;\left(\frac{\partial \gamma_{1}}{\partial x}+\nu \;\frac{\partial \gamma_{2}}{\partial y}-\frac{\left(1+\nu \right)\;\alpha_{t}}{h}\;T_{2}\right)=0,$$

Edges y=0 and y=b  (x∈(0,a), τ>0):

(6) $$w=0,\;\gamma_{1}=0,\;M_{y}=D\;\left(\frac{\partial \gamma_{2}}{\partial y}+\nu \;\frac{\partial \gamma_{1}}{\partial x}-\frac{\left(1+\nu \right)\;\alpha_{t}}{h}\;T_{2}\right)=0,$$

All four edges (τ>0):

(7) $$T_{1}=0,\;T_{2}=0,$$

Initial conditions (x∈(0,a), y∈(0,b)):

(8) $$T_{1}\left(x,y,0\right)=T_{1}^{0}\left(x,y\right),\;T_{2}\left(x,y,0\right)=T_{2}^{0}\left(x,y\right).$$

Here $D=E{\left(2h\right)}^{3}/\left[12\left(1-\nu^{2}\right)\right]$ is the flexural rigidity of the plate, *E* is Young’s modulus, and $M_{x}$ and $M_{y}$ are the bending moments per unit length about the *y*- and *x*-axes, respectively. Conditions (5) and (6) express the zero deflection, zero tangential rotation, and zero bending moment at each simply supported edge. Condition (7) prescribes zero temperature characteristics at all edges.

## 3. Solution Method

### 3.1. Thermal Problem

The heat conduction system (2) subject to the boundary conditions (7) is solved by applying the finite double Fourier sine transform in *x* and *y*. The temperature characteristics $T_{1}$ and $T_{2}$ are expanded as

(9) $$\left\{T_{1},\;T_{2}\right\}\left(x,y,\tau \right)=\sum_{m=1}^{\infty }\sum_{n=1}^{\infty }\left\{T_{1mn}\left(\tau^{\prime }\right),\;T_{2mn}\left(\tau^{\prime }\right)\right\}\sin\frac{\pi m}{a}x\;\sin\frac{\pi n}{b}y,$$

where $\tau^{\prime }=\left(\kappa /h^{2}\right)\;\tau$ is the dimensionless time. Substituting (9) into (2) reduces the thermal problem to the following system of first-order ordinary differential equations for the Fourier coefficients:

(10) $$\begin{array}{cc} \frac{dT_{1mn}}{d\tau^{\prime }}+g_{1}\;T_{1mn}+g_{2}\;T_{2mn}\; & =f_{1mn}\left(\tau^{\prime }\right),\\ \frac{dT_{2mn}}{d\tau^{\prime }}+g_{3}\;T_{1mn}+g_{4}\;T_{2mn}\; & =f_{2mn}\left(\tau^{\prime }\right), \end{array}$$

with constant coefficients

(11) $$\begin{array}{cc} \; & g_{1}=\mu_{m}^{2}+\mu_{n}^{2}+{\mathrm{Bi}}_{1},\;g_{2}={\mathrm{Bi}}_{2},\\ \; & g_{3}=3\;{\mathrm{Bi}}_{2},\;g_{4}=\mu_{m}^{2}+\mu_{n}^{2}+3\left(1+{\mathrm{Bi}}_{1}\right), \end{array}$$

and forcing terms

$$f_{1mn}=Q_{1mn}\;f_{1}^{\tau }\left(\tau^{\prime }\right),\;f_{2mn}=Q_{2mn}\;f_{2}^{\tau }\left(\tau^{\prime }\right),$$

where

$$\mu_{m}=\frac{\pi mh}{a},\;\mu_{n}=\frac{\pi nh}{b},\;{\mathrm{Bi}}_{i}=\varepsilon_{i}^{t}\;h^{2},\;\left(i=1,2\right).$$

### 3.2. Laplace Transform Solution

System (10) is solved analytically by the Laplace transform, with the initial conditions (8) imposed on the Fourier coefficients. The solution reads

(12) $$T_{1mn}=\sum_{j=1}^{2}\frac{{\left(-1\right)}^{j+1}}{p_{2}-p_{1}}\left[\left(p_{j}-g_{4}\right)\;Q_{1mn}\;Z_{1j}\left(\tau^{\prime }\right)+g_{2}\;Q_{2mn}\;Z_{2j}\left(\tau^{\prime }\right)+\left(\left(p_{j}-g_{4}\right)\;T_{1mn}^{0}+g_{2}\;T_{2mn}^{0}\right)e^{-p_{j}\tau^{\prime }}\right],$$

(13) $$T_{2mn}=\sum_{j=1}^{2}\frac{{\left(-1\right)}^{j+1}}{p_{2}-p_{1}}\left[\left(p_{j}-g_{1}\right)\;Q_{2mn}\;Z_{2j}\left(\tau^{\prime }\right)+g_{3}\;Q_{1mn}\;Z_{1j}\left(\tau^{\prime }\right)+\left(\left(p_{j}-g_{1}\right)\;T_{2mn}^{0}+g_{3}\;T_{1mn}^{0}\right)e^{-p_{j}\tau^{\prime }}\right],$$

where the eigenvalues of the coefficient matrix are

(14) $$p_{j}=\frac{g_{1}+g_{4}}{2}+{\left(-1\right)}^{j}\sqrt{\frac{{\left(g_{1}-g_{4}\right)}^{2}}{4}+g_{2}\;g_{3}},\;j=1,2,$$

the Fourier coefficients of the spatial data are

(15) $$\left\{Q_{1mn},\;Q_{2mn},\;T_{1mn}^{0},\;T_{2mn}^{0}\right\}=\frac{4}{ab}\int_{0}^{a}\;\int_{0}^{b}\left\{f_{1}^{z},\;f_{2}^{z},\;T_{1}^{0},\;T_{2}^{0}\right\}\left(x,y\right)\;\sin\frac{\pi m}{a}x\;\sin\frac{\pi n}{b}y\;dx\;dy,$$

and the convolution integrals encoding the time history of the thermal loading are

(16) $$Z_{ij}\left(\tau^{\prime }\right)=\int_{0}^{\tau^{\prime }}f_{i}^{\tau }\left(u\right)\;e^{-p_{j}\left(\tau^{\prime }-u\right)}\;du,\;i,j=1,2.$$

### 3.3. Thermoelasticity Problem

The system of thermoelasticity differential Equation (3) is also solved using the finite Fourier transform method. Then the generalized displacements are of the form

(17) $$\begin{array}{cc} w & =\sum_{m=1}^{\infty }\sum_{n=1}^{\infty }W_{mn}\sin\frac{\pi m}{a}x\;\sin\frac{\pi n}{b}y,\\ \gamma_{1} & =\sum_{m=1}^{\infty }\sum_{n=1}^{\infty }\Gamma_{1mn}\cos\frac{\pi m}{a}x\;\sin\frac{\pi n}{b}y,\\ \gamma_{2} & =\sum_{m=1}^{\infty }\sum_{n=1}^{\infty }\Gamma_{2mn}\sin\frac{\pi m}{a}x\;\cos\frac{\pi n}{b}y, \end{array}$$

and the system of differential Equation (3) will be transformed into a system of algebraic equations for determining the Fourier coefficients $W_{mn},\Gamma_{1mn},\Gamma_{2mn}$, which is written in matrix form

(18) $$\left(\begin{array}{ccc} m_{11} & m_{12} & m_{13}\\ m_{12} & m_{22} & m_{23}\\ m_{13} & m_{23} & m_{33} \end{array}\right)\left(\begin{array}{c} W_{mn}\\ \Gamma_{1mn}\\ \Gamma_{2mn} \end{array}\right)=\left(\begin{array}{c} r_{1}\\ r_{2}\\ r_{3} \end{array}\right)T_{2mn},$$

where

$$m_{11}=k^{\prime }\left(\mu_{m}^{2}+\mu_{n}^{2}\right);m_{12}=k^{\prime }\mu_{m};m_{13}=k^{\prime }\mu_{n};$$

$$m_{22}=\frac{h^{2}}{3}\left(\mu_{n}^{2}+\frac{2}{1-\nu }\mu_{m}^{2}\right)+k^{\prime };m_{23}=\frac{h^{2}}{3}\frac{1+\nu }{1-\nu }\mu_{m}\mu_{n};m_{33}=\frac{h^{2}}{3}\left(\mu_{m}^{2}+\frac{2}{1-\nu }\mu_{n}^{2}\right)+k^{\prime };$$

$$r_{1}=0;r_{2}=-\frac{2h}{3}\frac{1+\nu }{1-\nu }\mu_{m}\alpha_{t};r_{3}=-\frac{2h}{3}\frac{1+\nu }{1-\nu }\mu_{n}\alpha_{t}.$$

From the system (18), the solution is obtained:

(19) $$\left(\begin{array}{c} W_{mn}\\ \Gamma_{1mn}\\ \Gamma_{2mn} \end{array}\right)=\frac{1}{\left|M\right|}\sum_{i=1}^{3}\left[\left(\begin{array}{c} r_{i}A_{i1}\\ r_{i}A_{i2}\\ r_{i}A_{i3} \end{array}\right)T_{2mn}\right],$$

where $\left|M\right|$ is the determinant of the matrix ${\left(m_{ij}\right)}_{3\times 3}$, and $A_{ij}$ are the algebraic complements of the elements of this matrix.

Given the known generalized displacements and temperature characteristics of the average surface, the temperature field and normal stresses at any point on the plate are found, respectively, using Equations (1) and (4).

### 3.4. Solutions for Temperature Characteristics as a Function of Time-Varying Ambient Temperature

The numerical study is carried out under symmetric convective conditions ($\alpha^{+}=\alpha^{-}=\alpha_{z}$) with a one-sided on-the-surface z=+h ambient temperature that rises exponentially from zero to a steady-state value $t^{*}$:

(20) $$t_{z}^{+}\left(x,y,\tau \right)=t^{*}\;\left(1-e^{-\beta^{*}\tau }\right),$$

while on the surface z=−h, the temperature is maintained at zero $t_{z}^{-}\left(x,y,\tau \right)=0$, $T_{1}^{0}=T_{2}^{0}=0$, accordingly.

Here, $t^{*}$ is the specified maximum ambient temperature, and the parameter $\beta^{*}$ determines the rate at which the ambient temperature rises to the specified value $t^{*}$. For a material with λ=6.1 W/(m K) and h=1 mm, the characteristic thermal diffusion time is $\tau_{diff}=\frac{h^{2}}{\kappa }\approx 0.07$ s, which corresponds to an AMR cycle frequency of the order of 1–15 Hz; the value $\beta^{*}=1$ corresponds to a rapid heating regime commensurate with $\tau_{diff}$.

Substituting (20) into (15) and (16) yields the following closed-form expressions for the Fourier coefficients and convolution integrals:

(21) $$Q_{1mn}=\frac{2\;t^{*}\;\mathrm{Bi}}{\pi^{2}mn}\left(1-\cos\pi m\right)\left(1-\cos\pi n\right),\;Q_{2mn}=3\;Q_{1mn},$$

where $\mathrm{Bi}=\alpha_{z}h/\lambda$ is the Biot number, and

(22) $$Z_{ij}\left(\tau^{\prime }\right)=\frac{1}{p_{j}}\;\left(1-e^{-p_{j}\tau^{\prime }}\right)-\frac{1}{p_{j}-\beta^{*}}\;\left(e^{-\beta^{*}\tau^{\prime }}-e^{-p_{j}\tau^{\prime }}\right),\;i,j=1,2.$$

## 4. Numerical Results and Discussion

### 4.1. Material Data and Dimensionless Parameters

The results for the plate under consideration, made of two materials, are compared. The first is a magnetocaloric ferromagnetic alloy of the La(Fe,Co,Si)_13_ type, whose negative coefficient of thermal expansion reflects the anomalous behavior of the crystal lattice near the Curie point (see Section 1). The adopted value $\alpha_{t}=-26.1\times 10^{-6}\;K^{-1}$ corresponds to the peak negative value of the coefficient of thermal expansion of the La(Fe,Co,Si)_13_ alloy in the phase transition zone near $T_{C}$ and models the most critical scenario of thermomechanical loading.

$$E=130\;\mathrm{GPa},\;\nu =0.30,\;\alpha_{t}=-26.1\times 10^{-6}\;K^{-1},\;\lambda =6.1\;W\;m^{-1}K^{-1}.$$

The second is structural carbon steel, serving as a reference material with well-characterized, monotonically varying thermoelastic properties

$$E=210\;\mathrm{GPa},\;\nu =0.30,\;\alpha_{t}=12\times 10^{-6}\;K^{-1},\;\lambda =40\;W\;m^{-1}K^{-1}.$$

Unless otherwise stated, the remaining parameters are fixed at

$$a/b=1,\;h/b=0.05,\;\mathrm{Bi}=1,\;\beta^{*}=1,\;k^{\prime }=5/6.$$

Results are presented at the plate center (a/2,b/2) in terms of the following dimensionless quantities evaluated at the outer surface z=h:

$$w^{\prime }=\frac{w}{h\;t^{*}\alpha_{t}},\;t^{\prime }=\frac{t(h)}{t^{*}},\;\sigma_{1}^{\prime }=\frac{\sigma_{x}\left(h\right)}{E\alpha_{t}t^{*}},\;\sigma_{2}^{\prime }=\frac{\sigma_{y}\left(h\right)}{E\alpha_{t}t^{*}}.$$

### 4.2. Discussion of Results

**Figure 1:**
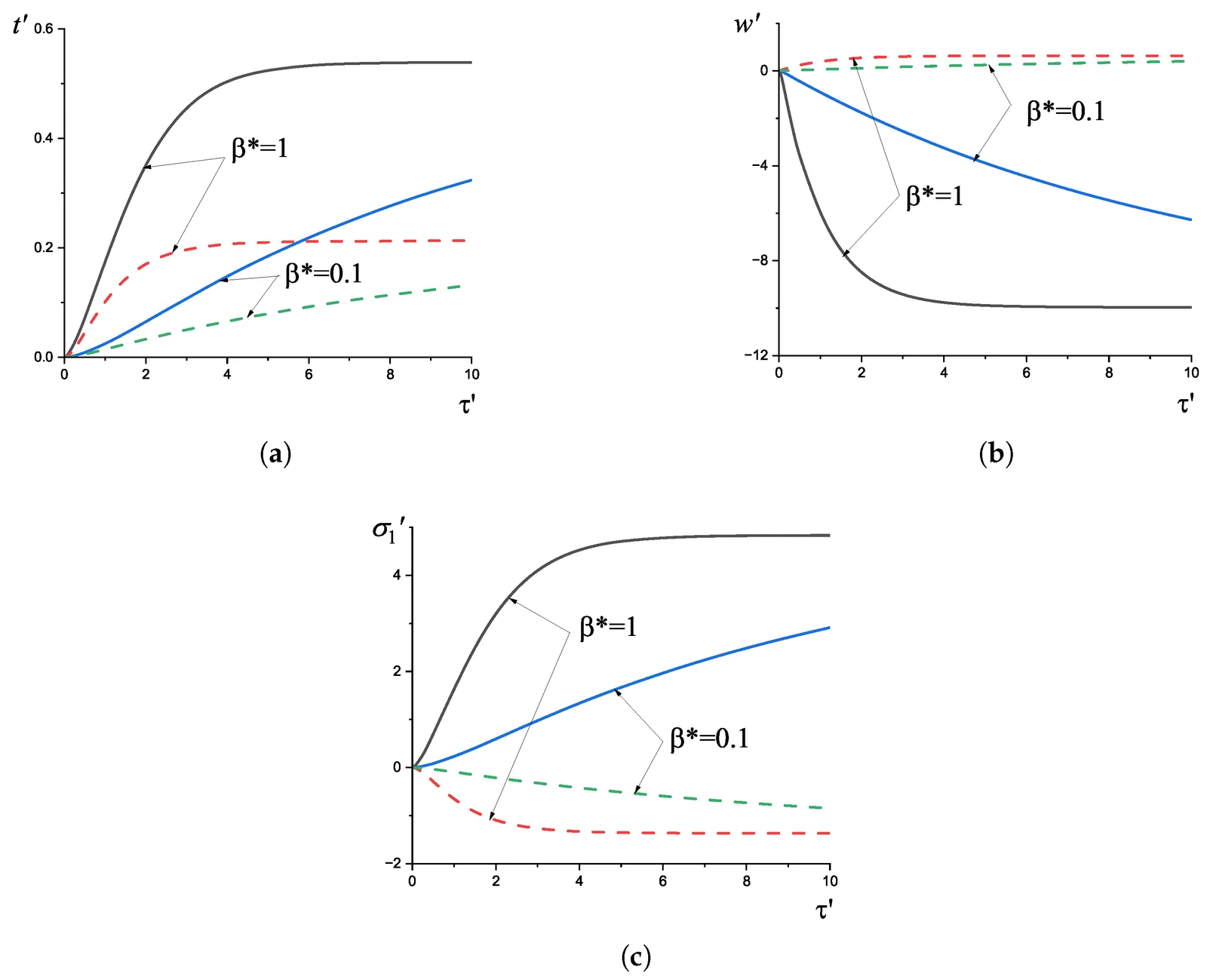
Time evolution of the dimensionless quantities at the plate center (a/2,b/2) for two values of the ambient temperature rise rate: $\beta^{*}=0.1$ (slow heating) and $\beta^{*}=1$ (fast heating) for the magnetocaloric ferromagnetic alloy (solid lines), and the corresponding results for carbon steel (dashed lines). (**a**) Dimensionless temperature $t^{\prime }$ versus dimensionless time $\tau^{\prime }$. (**b**) Dimensionless deflection $w^{\prime }$ versus dimensionless time $\tau^{\prime }$. (**c**) Dimensionless normal stress $\sigma_{1}^{\prime }$ versus dimensionless time $\tau^{\prime }$.

Figure 1 shows the time evolution of $t^{\prime }$, $w^{\prime }$, and $\sigma_{1}^{\prime }$ for two values of the heating rate parameter: $\beta^{*}=0.1$ (slow heating) and $\beta^{*}=1$ (fast heating). A higher value of $\beta^{*}$ accelerates the rise of both temperature and stress, and the transient peak stress in the ferromagnetic alloy differs significantly in sign from that in carbon steel. The results indicate a qualitatively different nature of the stress state in the two materials under identical thermal conditions: in the ferromagnetic plate, the normal stresses are tensile, whereas in the carbon steel plate, they are compressive. This change in the sign of the stresses is explained by the anomalous thermomechanical response of the magnetocaloric alloy: the negative coefficient of thermal expansion causes the material to contract upon heating, which, under free-edge boundary conditions, manifests as tensile stress, whereas the typical thermal response of carbon steel generates compressive stresses.

**Figure 2:**
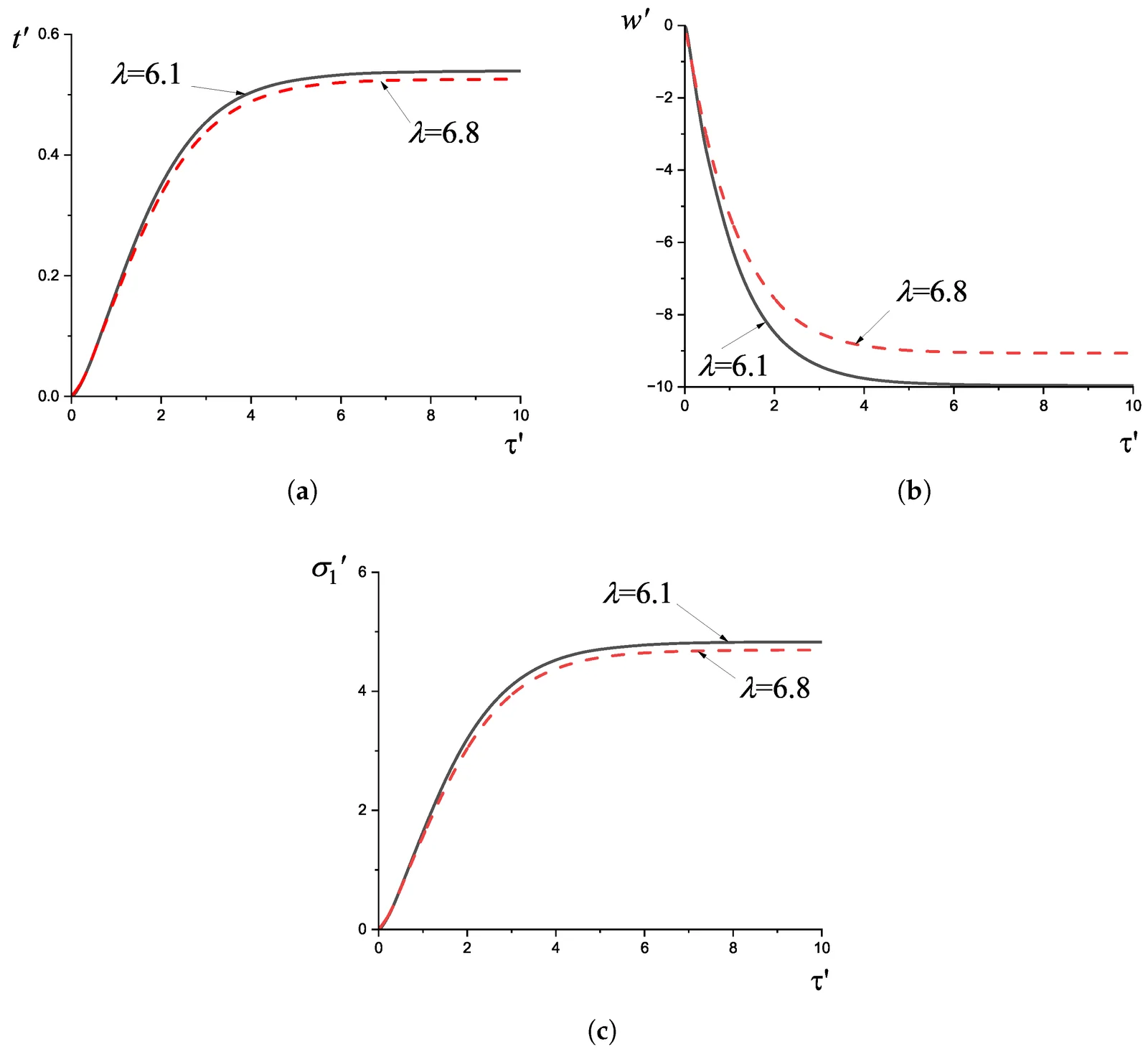
Time evolution of the dimensionless quantities at the plate center (a/2,b/2) for two values of the thermal conductivity of the magnetocaloric ferromagnetic alloy: $\lambda =6.1\;W\;m^{-1}K^{-1}$ (solid lines) and $\lambda =6.8\;W\;m^{-1}K^{-1}$ (dashed lines). (**a**) Dimensionless temperature $t^{\prime }$ versus dimensionless time $\tau^{\prime }$. (**b**) Dimensionless deflection $w^{\prime }$ versus dimensionless time $\tau^{\prime }$. (**c**) Dimensionless normal stress $\sigma_{1}^{\prime }$ versus dimensionless time $\tau^{\prime }$.

Figure 2 compares the same three quantities for two values of thermal conductivity of the ferromagnetic alloy (λ=6.1 and $6.8\;W\;m^{-1}K^{-1}$), illustrating the sensitivity of the stress field to the heat transfer characteristics of the magnetocaloric material under consideration.

**Figure 3:**
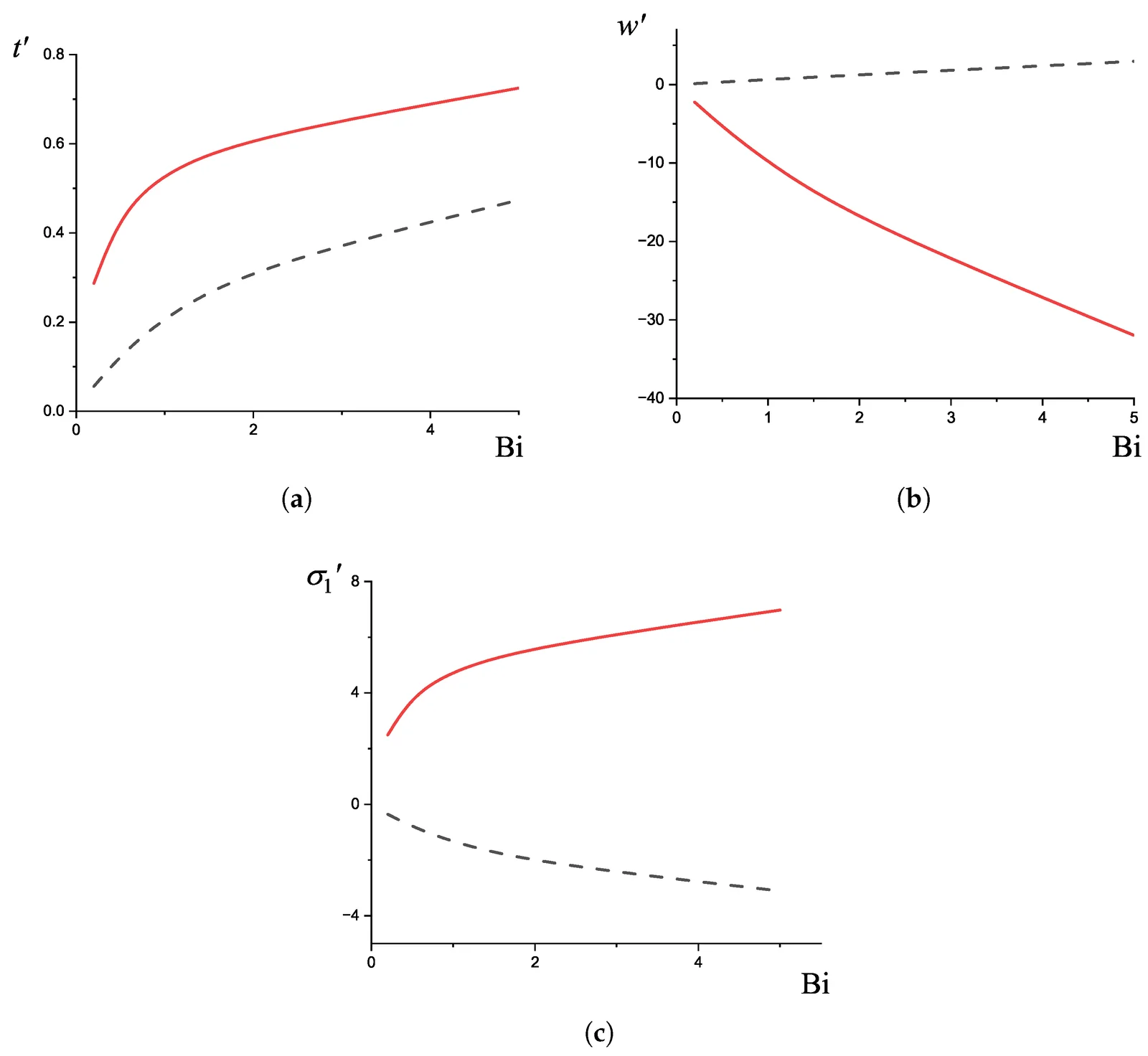
Dependence of the dimensionless quantities at the plate center (a/2,b/2) on the Biot number Bi for the magnetocaloric ferromagnetic alloy (solid lines) and carbon steel (dashed lines). (**a**) Dimensionless temperature $t^{\prime }$ versus Bi. (**b**) Dimensionless deflection $w^{\prime }$ versus Bi. (**c**) Dimensionless normal stress $\sigma_{1}^{\prime }$ versus Bi.

Figure 3 shows the dependence of $t^{\prime }$, $w^{\prime }$, and $\sigma_{1}^{\prime }$ on the Biot number Bi, which controls the intensity of convective heat exchange. An increase in Bi intensifies the surface heat flux and increases the thermal gradient across the thickness. This leads to a nonlinear increase in tensile stresses in the ferromagnetic plate and compressive bending stresses in the carbon plate.

**Figure 4:**
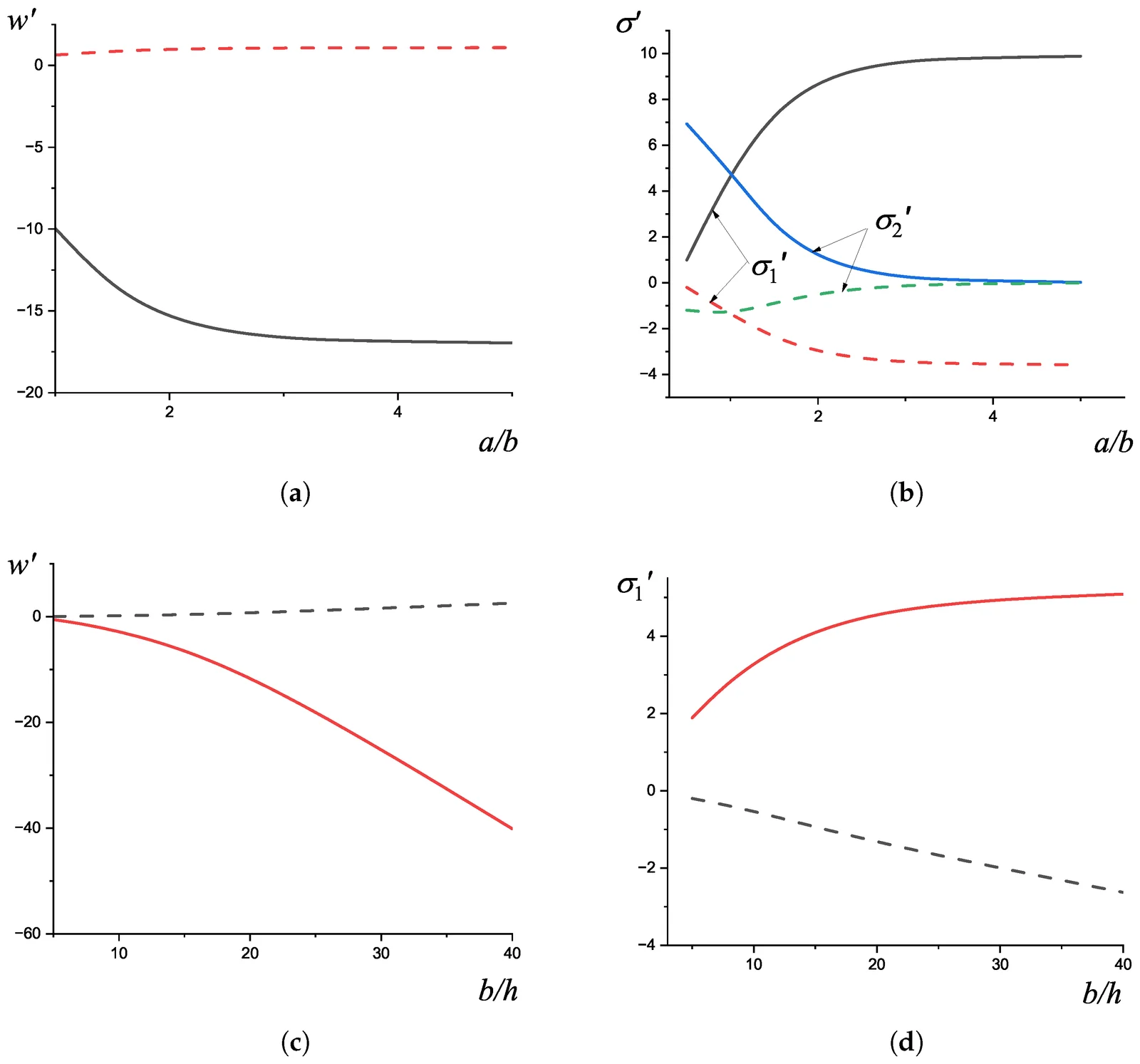
Influence of the plate geometry on the dimensionless quantities at the plate center (a/2,b/2) for the magnetocaloric ferromagnetic alloy (solid lines) and carbon steel (dashed lines). (**a**) Dimensionless deflection $w^{\prime }$ versus aspect ratio a/b. (**b**) Dimensionless normal stresses $\sigma_{1}^{\prime }$ and $\sigma_{2}^{\prime }$ versus aspect ratio a/b. (**c**) Dimensionless deflection $w^{\prime }$ versus thickness ratio b/h. (**d**) Dimensionless normal stress $\sigma_{1}^{\prime }$ versus thickness ratio b/h.

Figure 4 illustrates the influence of the plate geometry: panels (a,b) display $w^{\prime }$, $\sigma_{1}^{\prime }$, and $\sigma_{2}^{\prime }$ as functions of the aspect ratio a/b, while panels (c,d) show their dependence on the thickness ratio b/h. The results confirm that the stress state of the magnetocaloric plate is considerably more sensitive to geometric parameters than that of the carbon steel plate. This dependence has practical implications for the sizing of AMR plate stacks.

To assess the strength of the plate under consideration, the dimensional stress $\sigma_{x}$ is determined based on the calculated dimensionless stress $\sigma_{1}^{\prime }$. Taking $t^{*}$ as the adiabatic temperature change $\Delta T_{ad}$ characteristic of the La(Fe,Co,Si)_13_ alloy—which ranges from approximately 2 to 10 K for field changes of 0 to 2 T—the computed tensile stress at the plate center reaches $\sigma_{x}=E\cdot \left|\alpha_{t}\right|\cdot \Delta T_{ad}\cdot \sigma_{1}^{\prime }\approx 15.4\;\Delta T_{ad}$ MPa. For $\Delta T_{ad}=5$ K this yields $\sigma_{x}\approx 77$ MPa, while for $\Delta T_{ad}=10$ K the value rises to approximately 154 MPa. The latter exceeds the lower bound of the flexural fracture strength of 130–170 MPa measured by Wang et al. [7] on La(Fe,Mn,Si)_13_ plates of geometry identical to those used in AMR devices.

These estimates suggest that convective thermal loading contributes a stress of the order of 10–20% of the fracture strength reported for defect-free plates. The sensitivity of $\sigma_{1}^{\prime }$ to the plate geometry demonstrated in Figure 4 therefore has direct engineering implications: reducing b/h (increasing relative thickness) lowers the bending stress and widens the safety margin against fracture initiation at pre-existing cracks.

## 5. Conclusions

In this paper, based on the first-order equations of shear deformation theory, a methodology is developed for investigating the stress–strain state of an isotropic rectangular plate with freely supported edges, which is heated by the environment through heat exchange across its front surface. A closed-form analytical solution is obtained using the methods of trigonometric Fourier series and the Laplace transform, which allows for an effective description of the transient distribution of the temperature field, normal displacements, and stresses across the thickness and within the plane of the plate. A numerical comparative analysis was performed of the studied quantities in a ferromagnetic plate exhibiting the magnetocaloric effect, based on La(Fe,Si)_13_-type alloys, and in a carbon steel plate under conditions where the ambient temperature rises to a specified constant value. The dependencies of the studied quantities on the rate of ambient temperature rise $\beta^{*}$, time, the heat transfer coefficient Bi, the thermal conductivity coefficient of the ferromagnetic material λ, and the geometric parameters of the plate are illustrated graphically.

The results obtained can be directly applied to the analysis of the stress state of thin-walled plates with freely supported edges. In particular, the developed methodology naturally extends to the problem of evaluating the thermomechanical reliability of AMR plate elements, whose working medium consists of ferromagnetic plates.

A comparison with carbon steel allows us to estimate the quantitative limit of the safety margin—that is, the extent to which the specific brittleness of the magnetocaloric material exceeds that of conventional structural steel under the same thermal loads. This is useful for engineers who select the geometry and thickness of regenerator plates to ensure efficient heat transfer between the magnetocaloric material and the heat transfer fluid in microchannels. The practical relevance of the research conducted in this work may serve as a first step toward evaluating the thermomechanical behavior of parallel-plate regenerators.

Thus, the analytical method presented here is not only theoretically sound and suitable for parametric analysis, but also provides a basis for predicting the mechanical reliability and service life of laminated ferromagnetic elements in magnetic cooling devices during the design phase.

## Data Availability

The original contributions presented in this study are included in the article. Further inquiries can be directed to the corresponding author.

## Abbreviations

The following abbreviations are used in this manuscript:

MCE	Magnetocaloric effect
MCM	Magnetocaloric material
FSDT	The first-order shear deformation theory
AMR	Active magnetic regenerators
